# Protein–Ligand CH−π Interactions: Structural Informatics, Energy Function Development, and Docking Implementation

**DOI:** 10.1021/acs.jctc.3c00300

**Published:** 2023-07-26

**Authors:** Yao Xiao, Robert J. Woods

**Affiliations:** Complex Carbohydrate Research Center, University of Georgia, Athens, Georgia 30602, United States

## Abstract

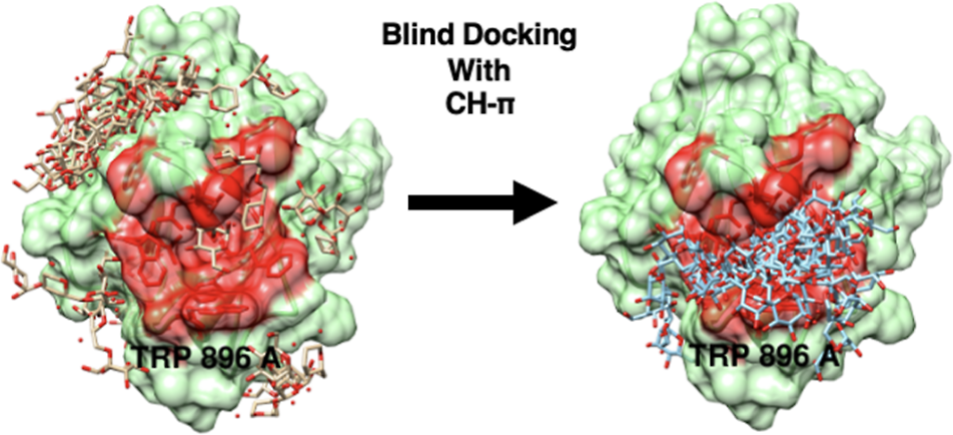

Here, we develop an empirical energy function based on quantum mechanical data for the interaction between methane and benzene that captures the contribution from CH−π interactions. Such interactions are frequently observed in protein–ligand crystal structures, particularly for carbohydrate ligands, but have been hard to quantify due to the absence of a model for CH−π interactions in typical molecular mechanical force fields or docking scoring functions. The CH−π term was added to the AutoDock Vina (AD VINA) scoring function enabling its performance to be evaluated against a cohort of more than 1600 occurrences in 496 experimental structures of protein–ligand complexes. By employing a conformational grid search algorithm, inclusion of the CH−π term was shown to improve the prediction of the preferred orientation of flexible ligands in protein-binding sites and to enhance the detection of carbohydrate-binding sites that display CH−π interactions. Last but not least, this term was also shown to improve docking performance for the CASF-2016 benchmark set and a carbohydrate set.

## Introduction

Protein–ligand interactions modulate innumerable physiological events, including, but not limited to, cell signaling,^1^ infection and disease onset,^2^ immune response,^3^ aging,^4^ cell death,^5^ etc. Inhibition of ligand binding is therefore a core mechanism of action of many therapeutic agents. Computational methods play central roles in the rational design of small molecules that inhibit protein–ligand binding, particularly aiding in the prediction of putative 3D structures and binding energies. In terms of classical computational studies, a crucial determinant of calculation accuracy is the quality of the force field, for energy minimization or molecular dynamics (MD) simulation, or energy predictor function, a.k.a. scoring function, for molecular docking.^6^ Docking is frequently employed when experimental methods such as X-ray crystallography are not applicable or for the screening of large virtual libraries of small molecules in the quest for a lead inhibitor molecule.^7,8^ While MD simulations may be employed to refine preliminary structures of complexes generated by docking, such refinement assumes that a reasonable structure was generated by docking. It is therefore of vital importance to ensure that docking scoring functions are as accurate as practicable. Although most scoring functions include approximations for common interactions, such as van der Waals and electrostatics, other noncovalent interactions, such as cation−π, anion−π, CH−π, and π–π, are generally overlooked, despite their prevalence in biomolecular systems. The CH−π interaction,^9^ specifically, is widely implicated in protein folding,^10^ carbohydrate recognition,^11−13^ and drug binding.^14^

In this paper, we present a structural analysis of CH−π interactions between aliphatic CH bonds and aromatic moieties present in the protein databank (PDB), develop a simple interaction energy function from quantum mechanical (QM) calculations on model CH−π complexes, and implement it in a version of AutoDock Vina^15^ (AD VINA) docking software augmented for application to carbohydrate ligands (VINA-CARB^16^). The experimental CH−π complexes in the PDB displayed strong geometric preferences, which enabled us to evaluate the accuracy of the theoretical CH−π energy function on a large experimental data set.

## Methods

### PDB Data Mining, Detection, and Geometric Analysis of Protein–Ligand CH−π Structures

Prefiltering of protein–ligand PDB structures to select only nonredundant protein chains was performed using the PISCES server^17^ as of January 2021. Further constraints included setting the maximum sequence percent identity to 95% to ensure that chains with highly similar sequences were considered only once. The maximum resolution was set to 2.5 Å, and the maximum *R* value was set to 0.3. Minimum and maximum chain length were set to 40 and 10,000 residues, respectively. A total of 37,843 chains were found.

**Figure 1:**
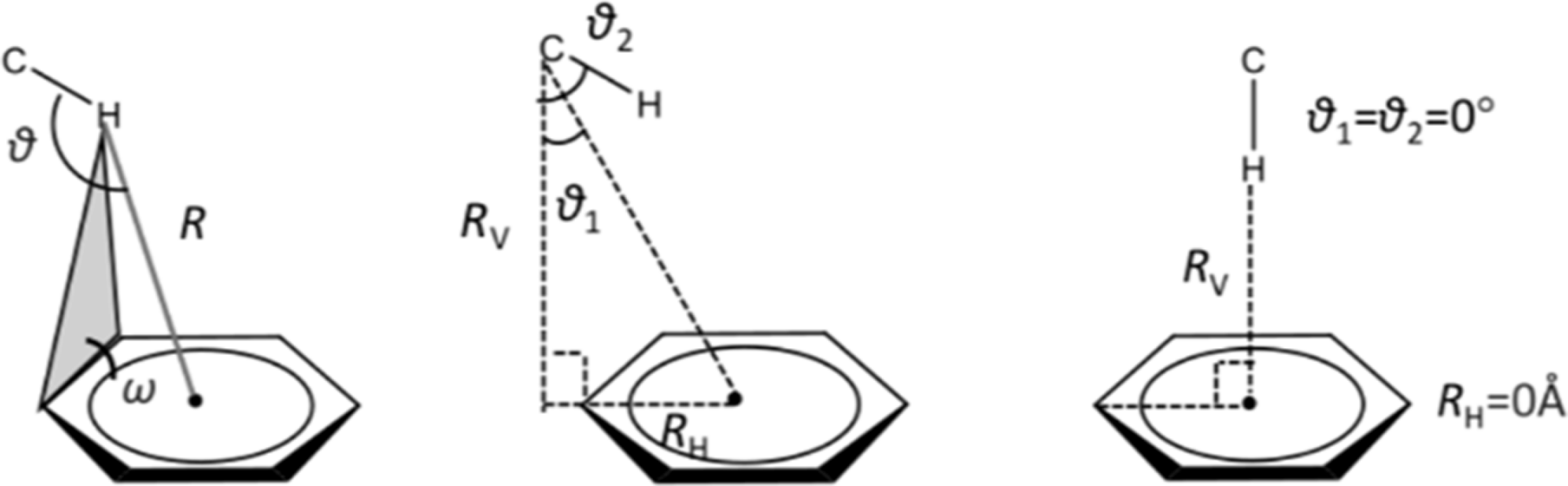
(Left) Geometric parameters defined by Kumar and Balaji^10^ and employed here. The ring centroid is indicated with a dot, and *R* represents the H-centroid distance. The C–H-centroid angle (θ) and the dihedral angle (ω) between the ring plane and the plane formed by the H and the two nearest ring carbon atoms are shown. Middle: two additional angular parameters (θ_1_ and θ_2_) and the horizontal (*R*_H_) and vertical (*R*_V_) offsets probed in the present study. (Right) Image of a perpendicular CH moiety above the phenyl ring.

For each chain obtained from the culling protocol, the corresponding entry was downloaded from the PDB. Hydrogen atoms were added to each structure using the AutodockTools^18^ utility script and processed into the PDBQT file format. Residues in which any atom had a partial occupancy were excluded from analysis, as were any residues with an average *B*-factor over 40.^10^ Aromatic rings in either the protein or the ligand were detected, and the coordinates of the ring centroid were computed. Distances between the aromatic ring centroid and all aliphatic protons in the corresponding binding partner molecule were computed. Following the CH−π analysis performed by Kumar and Balaji,^10^ CH−π interactions were defined as existing when the aliphatic H to centroid distance was ≤3.5 Å. For each such instance, the following geometric parameters were calculated: *H*_aliphatic_–centroid distance (*R*); *C*_aliphatic_–*H*_aliphatic_–entroid angle (θ); the dihedral angle between ring plane and the plane formed by the *H*_aliphatic_ and the two closest ring carbon atoms (ω); and the distance between *C*_aliphatic_ and ring centroid (Figure 1, left). Additionally, the following metrics were determined: the angle between the *C*_aliphatic_–centroid–ring normal (θ_1_) and the angle between the *C*_aliphatic_–*H*_aliphatic_–normal (θ_2_) (Figure 1, middle). Putative CH−π interactions involving methyl groups were not analyzed due to the unrestricted orientations of the protons.

### QM Calculation of Methane–Benzene Dimer Interaction Energies

**Figure 2:**
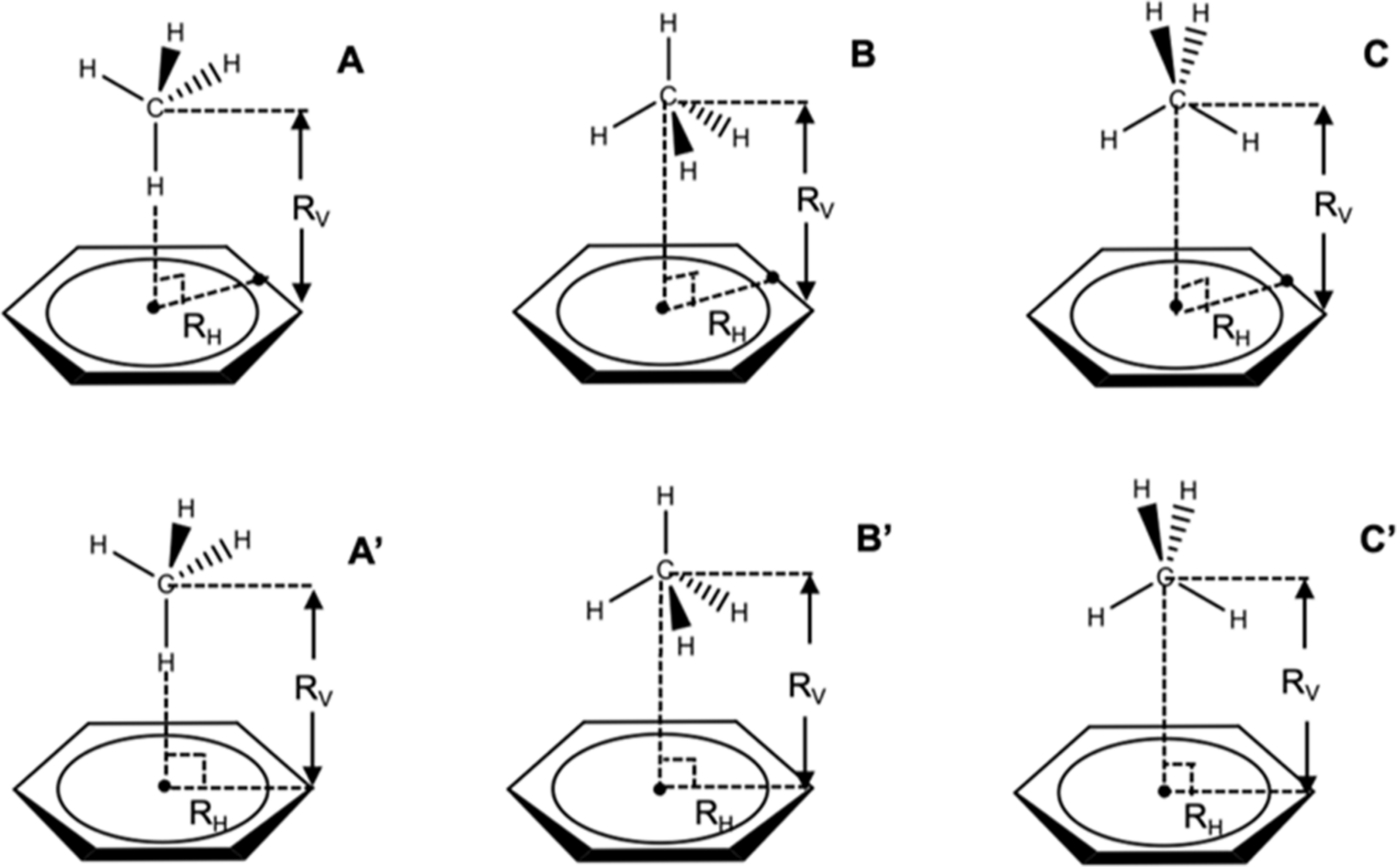
Six different methane-dimer geometries probed in our QM calculation.

Three-dimensional coordinates of methane and benzene were constructed and optimized separately with the Gaussian09^19^ program at the B3LYP/6-31G(d) level of theory. Methane–benzene dimer geometries were constructed using the optimized monomer coordinates (Figure 2). Two-dimensional surface scans for the six methane–benzene geometries (Figure 2) were performed as follows: (1) the vertical distance between the methane carbon and benzene ring center (*R*_V_) was constrained to values between 3.0 and 6.0 Å, with an increment of 0.1 Å between 3.0 and 4.2 Å and an increment of 0.2 Å between 4.2 and 6.0 Å and (2) the horizontal offset between the methane carbon and the benzene ring centroid (*R*_H_) was scanned from 0.0 to 1.4 Å with an increment of 0.1 Å along the vector defined by the ring centroid and a benzene carbon atom. Similarly, scanning was performed from 0.0 to 1.2 Å along the ring centroid to the benzene C–C bond midpoint vector. Additionally, at the equilibrium geometry (*R*_H_ = 0.0 Å), the scanning was extended to 6.0 Å. Single-point complexation energies for each dimer were computed at the MP2/aug-cc-pVDZ level of theory and corrected for basis set superposition error with the counterpoise method.^20^

### CASF-2016 Docking with AD VINA and Carbohydrate Docking with VINA-CARB

For focused docking, the grid box was set to the geometric center of each ligand, with box dimensions extending beyond the ligand by 8 Å in each Cartesian direction (−*x*, *x*; −*y*, *y*, and −*z*, *z*). Because AD VINA (and VINA-CARB) employs an 8 Å cutoff for computing intermolecular interactions, the final size of the grid box was thus sufficient to contain the entire binding site. For blind docking, the grid box was set to the geometric center of each protein, with box dimensions extending beyond the protein surface by 8 Å in each Cartesian direction. An exhaustiveness value of 12 was applied. Twenty ligand poses were produced per docking run. The maximum range of binding energy of all output models was set to 10 kcal/mol. Docking experiments were performed with varying weighting factor (*w*_CH−π_) values, ranging from 0.0 to 1.0, with an increment of 0.1. All docking experiments were repeated in triplicate with explicit random seed values of 10,000, 50,000, and 100,000 to enable statistical evaluations.

## Results

### PDB Data Mining, Detection, and Geometric Analysis of Protein–Ligand CH−π Structures

**Figure 3:**
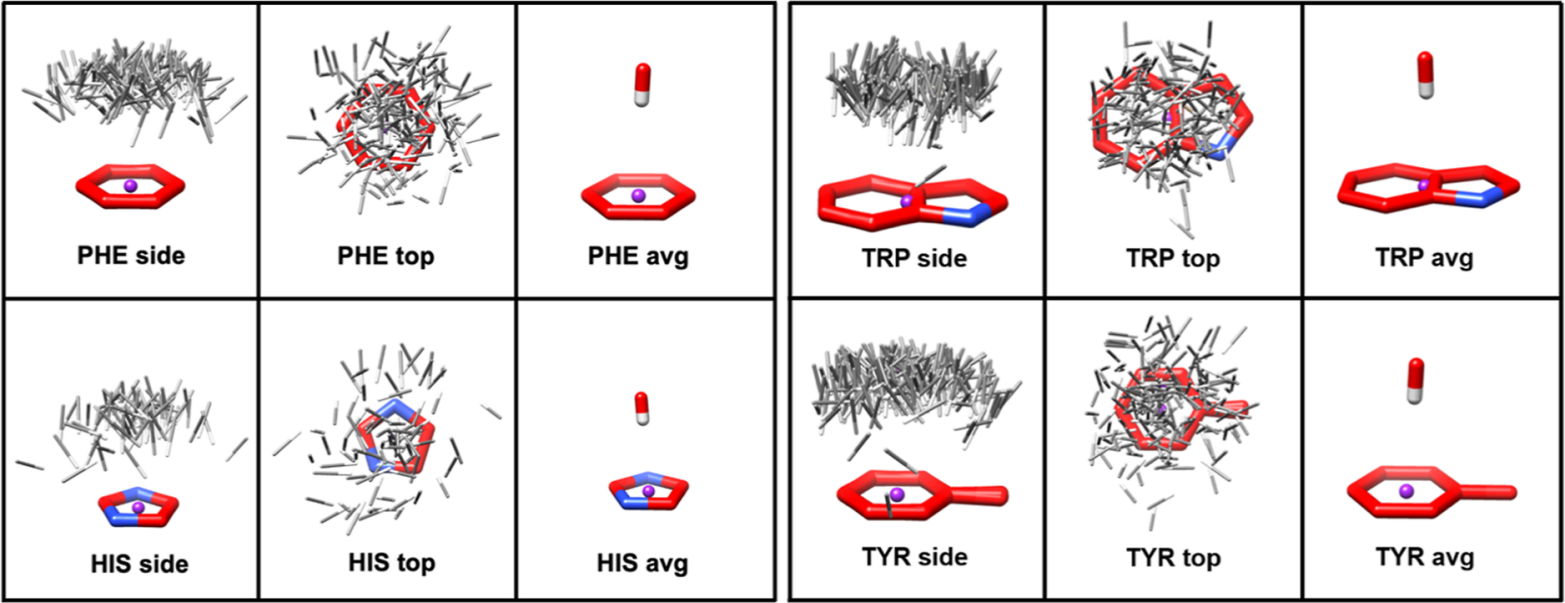
Examples of CH−π interactions culled from the PDB involving the aromatic rings of PHE (*n* = 173), HIS (*n* = 84), TRP (*n* = 297), and TYR (*n* = 192). The CH coordinates have been reflected so as to all be on the same face of the ring. Only the CH bonds are shown (carbon in gray and hydrogen in white). The centroid of each ring is shown in purple. The average CH positions are also shown.

**Figure 4:**
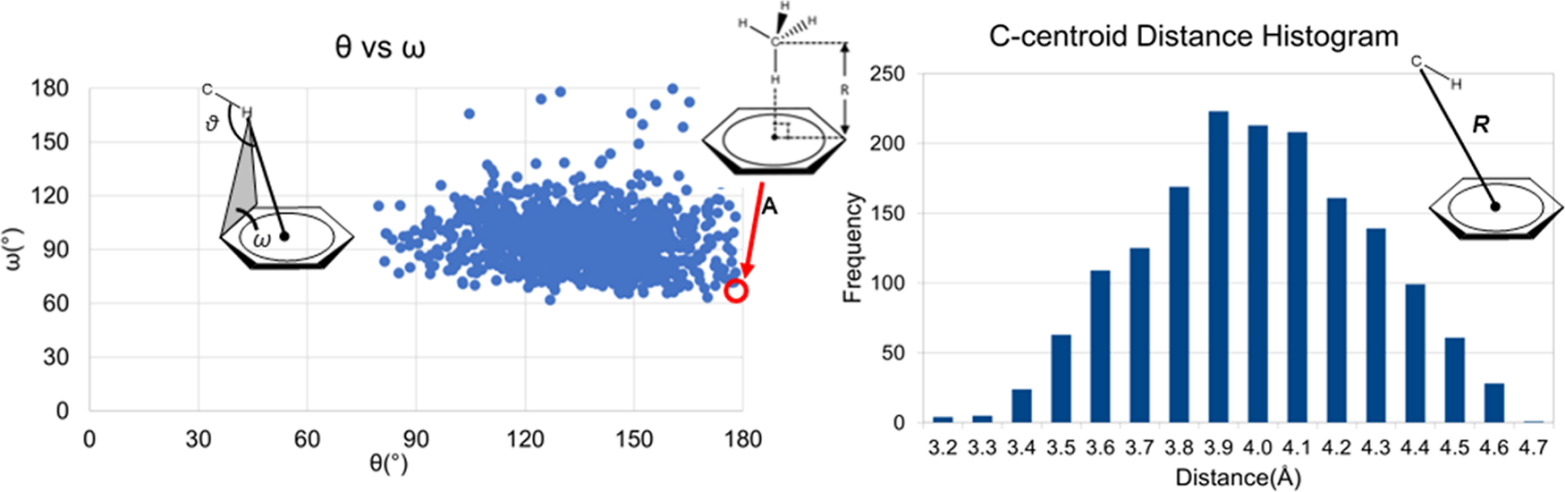
(Left) Scatter plot of θ and ω angle for the complete CH−π data set. The red circle corresponds to the canonical type A geometry (Figure 2) where θ = 180° and ω ≈ 60°. (Right) Histogram of the C-centroid-normal angle.

A total of 1635 instances of CH−π interactions were detected between a CH moiety and an aromatic ring in 496 PDB nonredundant structures, including interactions involving methyl groups (Table S1) or 1186 instances if methyl groups were not counted (Table S2). The relatively small number of PDB structures identified as containing CH−π interactions is primarily due to our desire to avoid inclusion of redundant examples, which was achieved by requiring that the sequence identity be less than 95%, a practice documented in an earlier protein–protein CH−π data mining study.^10^ Based on the uncertainty of hydrogen positions in methyl groups, putative CH−π interactions with methyl groups were excluded from consideration in any analysis involving hydrogen atoms. Shown in Figure 3 are the distributions of ligand CH groups interacting with the aromatic rings of PHE, HIS, TRP, and TYR. The locations of the CH bonds and their orientations relative to the rings display a very diffuse distribution. It is notable that the average position of the CH bonds is approximately directly above the ring centroid and perpendicular to the ring plane in all four aromatic amino acids, corresponding to a canonical type A geometry (Figure 2). Nevertheless, the actual distribution rarely samples such an ideal geometry. This can also be demonstrated from the relationship between the θ and ω angles (Figure 4, left), which shows that the region corresponding to type A geometry (θ = 180° and ω ≈ 60°) is not significantly populated.

The distribution of CH−π geometries, as characterized by the H-centroid distance and angles θ and ω, was comparable to the distribution from an analysis of CH−π interactions in the PDB reported by Kumar and Balaji^10^ (see Figure S1). The ω dihedral angle displayed a peak at approximately 100° (Figure S1, left), indicating that most hydrogen atoms are slightly displaced horizontally from the aromatic ring; a value of 90° would place the CH hydrogen atom directly above an edge of the ring. The tilt angle (θ_2_) in the CH−π PDB data set (Figure S1, right) populated a range of values with a mean of 38° ±19°, again indicating rarity of the canonical perpendicular orientation of the CH bond above the ring plane. Taken together, structural data from the PDB reveals that the CH bonds in CH−π interactions are broadly distributed over the face of the aromatic ring but display a well-defined vertical offset from the face of the ring with an average C-centroid distance of 3.9 ± 0.3 Å (Figure 4, right), which is consistent with data from previous theoretical studies that indicate approximately 3.8 Å for C-centroid distance.^22,23^

Although these interactions appear to be weak, their observed prevalence in crystal structures suggests that they frequently make a small but significant contribution to ligand – protein binding. As computational ligand docking software, such as AD VINA, is often used to predict ligand binding, we first sought to establish whether the VINA energy function was able to demonstrate the presence of CH−π stabilization. When applied to the experimentally observed interactions involving aromatic protein side chains (Figure 3), the VINA scoring function led to an average interaction energy of −0.1 ± 0.2 kcal/mol (Table S3) for each of the complexes in our data set (Figure S2). This result suggested that the current VINA scoring function failed to identify favorable CH−π interactions and led us to create a functional form for the CH−π energy that could be employed with the VINA scoring function.

### Hydrogen-Dependent CH−π Functional Form

**Figure 5:**
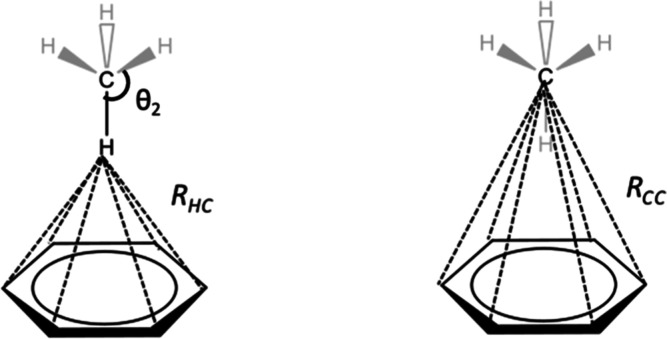
Illustration of the pairwise interatomic distances between the aliphatic proton (left, *R*_HC_) or the aliphatic carbon (right, *R*_CC_) and each of the aromatic ring carbon atoms which are employed additively to compute the hydrogen-dependent (1) and hydrogen-independent (eq 4) CH−π interaction energy, respectively. Atoms shown in gray are excluded from the calculation.

The molecular mechanical CH−π interaction energy was defined as the sum of the individual interactions between each aliphatic proton and each aromatic carbon ring atom (Figure 5, left). Following this approach, the maximum CH−π interaction energy (*E*_CH−π_) occurs when the aliphatic proton is directly above the ring centroid. A pairwise atomistic function has the additional advantage that the contribution from different ring atom types (C, N, O, etc.) may be defined, as needed. To incorporate the angular dependence of the orbital interactions between the C–H bond and the aromatic π system,^24,25^ each pairwise CH−π interaction was scaled by an angular decay component [*f*(θ_2_)]^26^ that depends on the tilt angle (θ_2_ in Figure 1, middle) between the C–H vector and ring normal (eq 1).
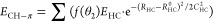
1where “*E*_HC_” is the maximum interaction energy of each H-aromatic carbon atom pair, “*R*_HC_” is the distance between that atom pair, “*R*^0^_HC_” is the optimal separation for that atom pair, and “*C*_HC_” controls the breadth of the curve. A cosine decay function, *f*(θ_2_), was chosen (eq 2) because it has a maximum when the C–H vector and ring normal are parallel, decaying to a value of 0 when they are perpendicular.
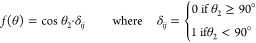
2The total interaction energy between an aliphatic CH and an aromatic system (*E*_CH*-*aromatic_) is thus the sum of *E*_CH−π_ and the energy computed from the uncorrected AD VINA scoring function (*E*_Vina_)
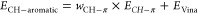
3where *w*_CH−π_ is an empirical weighting factor that scales the CH−π term and was set to 1 during curve fitting. Constants *E*_HC_, *R*^0^_HC_, and *C*_HC_ were derived by fitting eq 3 to QM data computed at the MP2/aug-cc-pVDZ//B3LYP/6-31G(d) level. Since AD VINA has a soft repulsion term that increases quadratically with overlap distance, it was not possible to accurately reproduce QM energies in the repulsive range.

**Figure 6:**
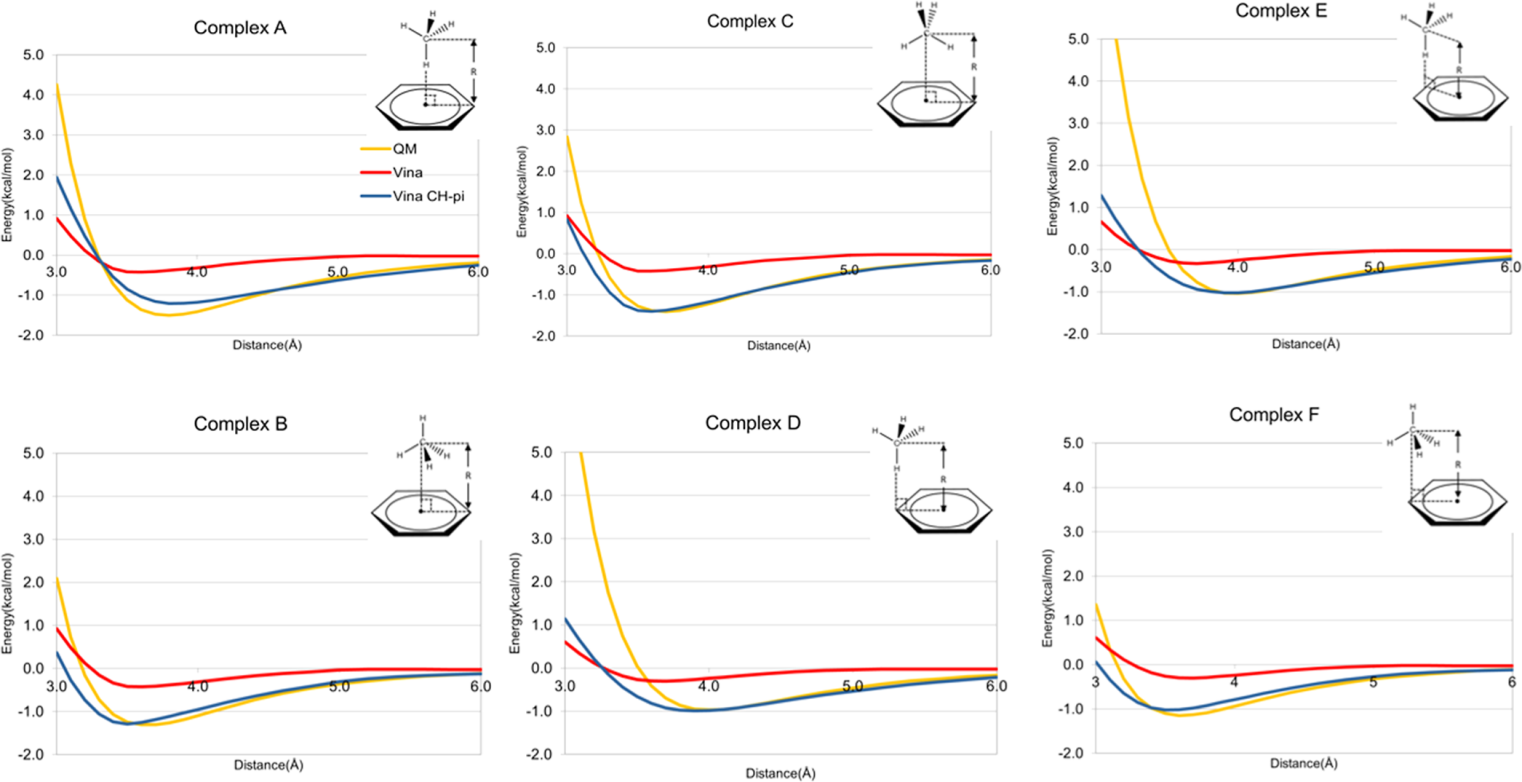
Methane–benzene dimer interaction energies at various vertical distances and six different dimer geometries. Six dimer geometries numbered A–F were present, with an illustration image at the top right of each figure. Horizontal axis, distance (Å), vertical axis, interaction energy, kcal/mol. Yellow line, MP2/aug-cc-pVDZ value. Red line, value from the unmodified AD VINA scoring function. Blue line, value from the unmodified AD VINA scoring function plus this new CH−π function.

Therefore, for each of the six methane–benzene geometries (Figure 6), fitting started at the point where the QM interaction energy becomes negative and ended when *R* = 6.0 Å. The starting *R* values for fitting QM data for complexes A–F were thus 3.3, 3.2, 3.3, 3.6, 3.6, and 3.2 Å. The function parameters were varied between *E*_HC_ = 0.10–0.50, *R*^0^_HC_ = 1.50–4.55, and *C*_HC_ = 0.05–2.00 at intervals of 0.05. For each combination of parameter values, the average rmsd between the predicted energies and the QM energies within the fitting range was computed. Once the combination resulting in the lowest average rmsd was determined, each parameter was then varied between ±0.05 around its current value at an interval of 0.01. The optimal values for the parameters in eq 1, namely, *E*_HC_ = 0.29, *R*^0^_HC_ = 3.58, and *C*_HC_ = 0.85, resulted in the lowest average rmsd between the QM and the empirical energies (0.164 kcal/mol) from the grid search (Figure 6).

At short intermolecular distances, the QM repulsion energies were markedly steeper than VINA. During the derivation of the CH−π function, the repulsion energy was dampened to be consistent with the soft repulsion employed in AutoDock VINA. The best fit to the empirical function resulted in an average optimal interaction distance of 3.7 ± 0.2 Å with an energy of −1.2 ± 0.2 kcal/mol, which can be compared to the corresponding QM values of 3.8 ± 0.2 Å and −1.2 ± 0.2 kcal/mol (Figure 6).

**Figure 7:**
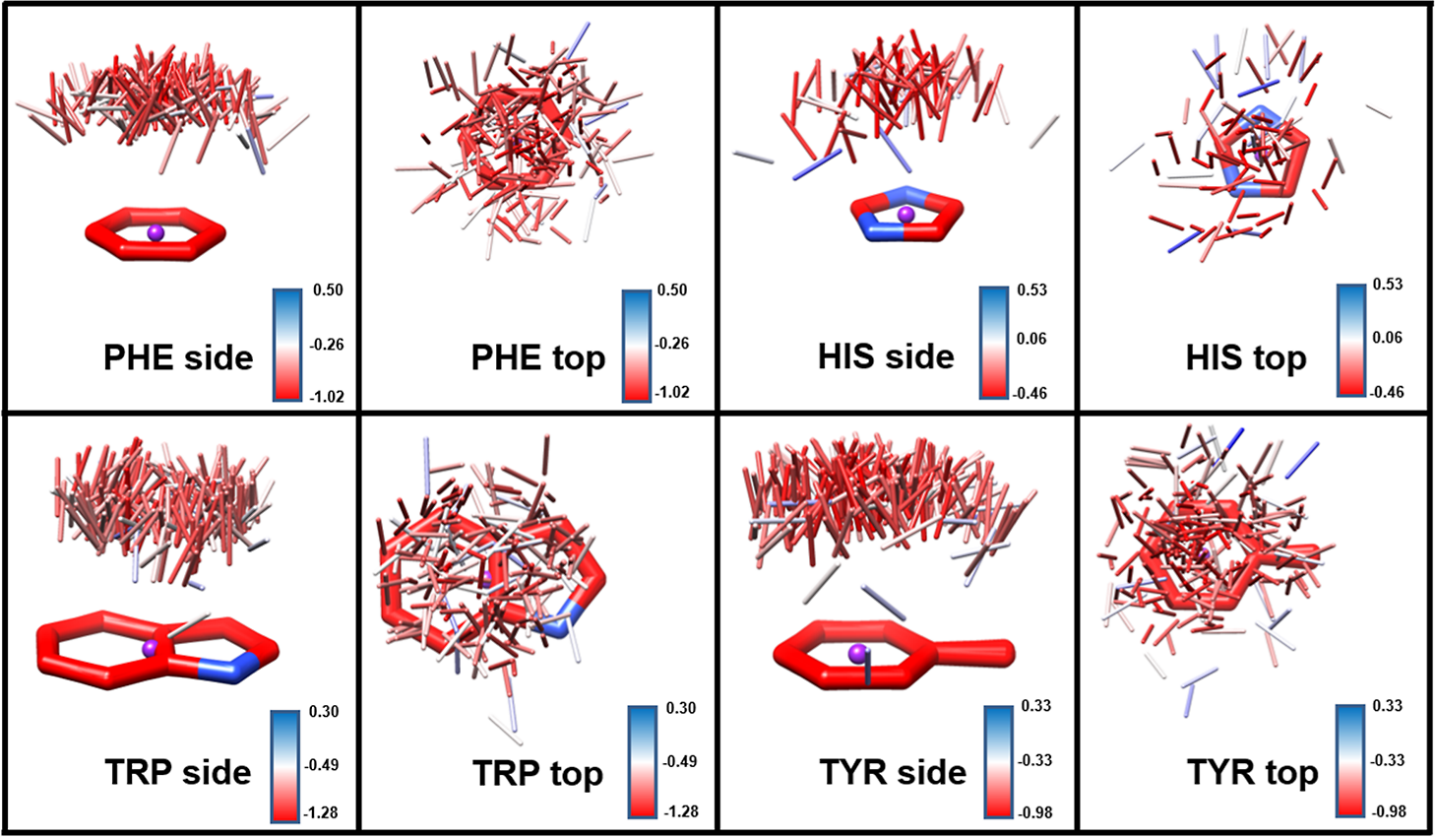
Examples of CH−π interactions culled from the PDB involving the aromatic ring of PHE, HIS, TRP, and TYR, colored by their interaction energy (VINA + CH−π). The centroid of each ring is shown in purple. Each accompanying red-white-blue color gradient describes the range of interaction energies in that image.

Having fit the empirical function to the QM data for model interactions, the function was then employed to predict the strengths of the experimentally observed CH−π interactions in Figure 3 (Figure 7). In contrast to the unmodified VINA function, which showed negligible interaction energies (average interaction energy of approximately −0.1 kcal/mol, Figure S2) for this data set, the average interaction energy for each of the complexes computed using VINA augmented with the CH−π function was PHE −0.7 ± 0.2 kcal/mol, HIS −0.2 ± 0.2 kcal/mol, TRP −0.8 ± 0.2 kcal/mol, and TYR −0.7 ± 0.2 kcal/mol. These values compare favorably to the experimentally determined interaction energy for *N*-acetylglucosamine with a PHE of −0.80 to −0.86 kcal/mol^27^.

**Figure 8:**
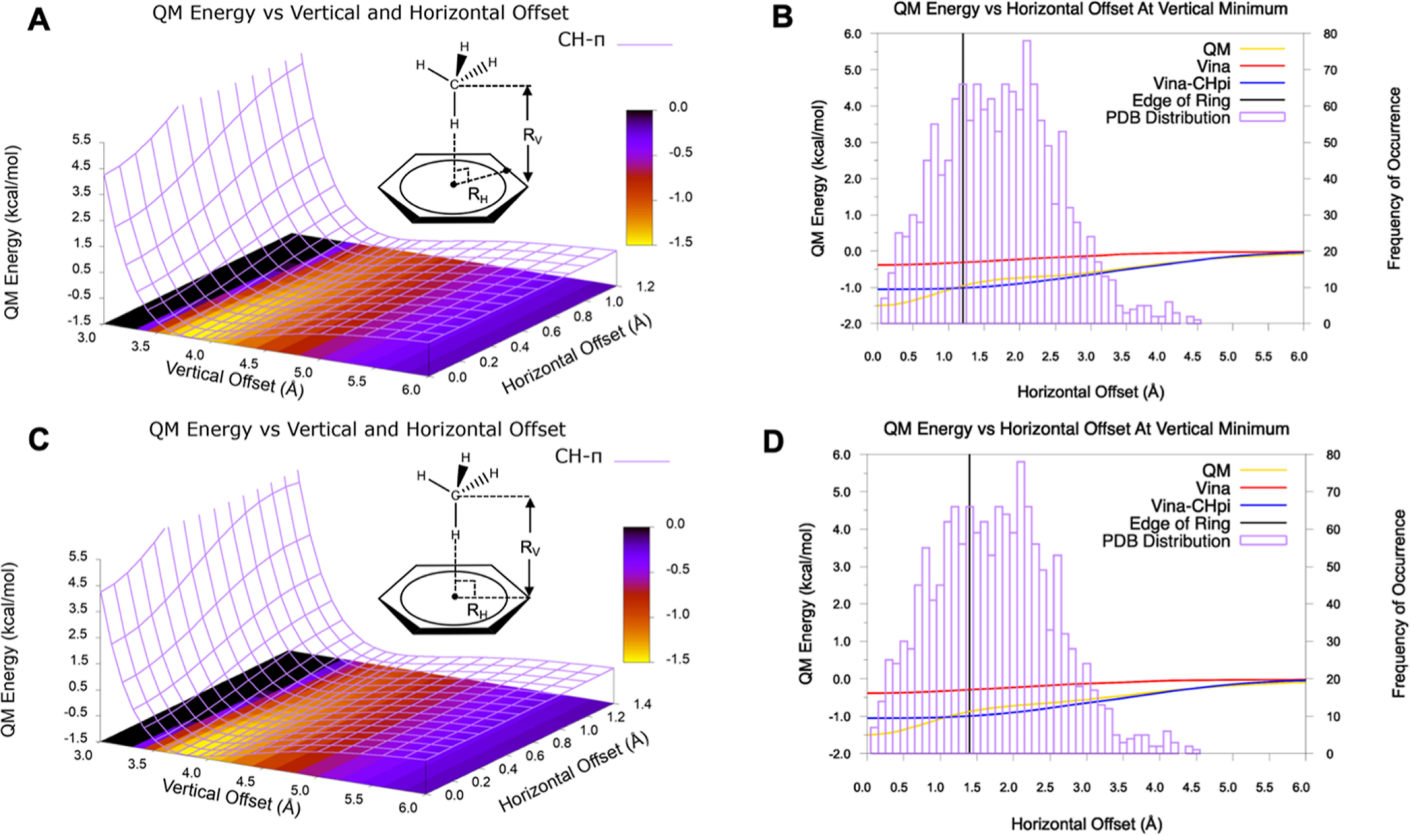
Methane–benzene dimer interaction energies for the type A geometry at various vertical and horizontal distances, and the histogram of the corresponding H-centroid horizontal offset values in the experimental structures. (A) QM energies of the dimer geometries with methane translated horizontally along the line between the ring centroid and the midpoint of a benzene C–C bond. (B) Trace through the 2D energy surface at a vertical separation (*R*_v_) of 3.8 Å showing the QM, VINA, and VINA energies plus CH−π energies. The histogram reports the frequency of experimental structures corresponding to the type A dimer geometry as a function of the horizontal offset (*R*_H_). (C,D) are same as (A,B) except that methane was horizontally translated along the ring centroid to the benzene atom vector.

The performance of the CH−π functional form was compared to QM energies computed for 2D surface scans for each of the six dimer geometries (Figures 2, 8, and S3), in which the vertical and horizontal offsets between the methane and benzene ring were varied (see Methods). It was observed for all dimer geometries that the magnitude of the QM energy decreased monotonically along the horizontal and vertical directions with a convergence to 0 at approximately 6.0 Å and a minimum with the methane located directly above the ring centroid. Regarding the canonical type A geometry, the QM energy showed a clear minimum at the ring centroid (−1.5 kcal/mol), whereas the Vina–CH−π energy was largely insensitive to the horizontal offset as long as the methane remained over the ring, giving rise to an interaction energy of −1.0 kcal/mol. In contrast, the interaction energy curve for AD VINA without the CH−π term was largely insensitive to the horizontal offset and gave rise to an interaction energy of approximately −0.4 kcal/mol (Figure 8B,D). Although the QM energies displayed a well-defined minimum for the methane directly above the ring centroid (*R*_H_ = 0.0 Å), the experimental distribution of CH interactions showed no such preference. Rather, the experimental structures were diffused over the entire ring surface, with the distribution suggesting a preference for alignments around the periphery of the ring (Figure 8B,D). It is noteworthy that the observed flatness of the Vina–CH−π interaction energy over the face of the ring appeared to be consistent with the experimental distribution for the corresponding interactions. Interestingly, for all other methane-dimer geometries, the QM and Vina–CH−π interaction energy curves were in agreement with each other, particularly as regards predicting a well-defined minimum with the methane directly above the ring centroid (Figure S3).

### Optimization of the CH−π Weighting in the Vina Scoring Function

The empirical function was fit to reproduce gas-phase QM data for model complexes. The optimal contribution (*w*_CH−π_) of this term to the VINA scoring function (eq 3) was determined based on its ability to reproduce the geometries of a set of 228 experimentally determined CH−π complexes. This data set contained structures with at least one CH−π instance satisfying θ_1_ < 35° and θ_2_ < 35° (Figure 1, middle). This cutoff criterion was employed to ensure that the development set contained relatively strong CH−π interactions. It should be noted that these structures were selected based on their conformity to the geometric definition of a CH−π interaction, instead of the presence of experimental data that proves the existence of CH−π interactions. Structures were subsequently examined in UCSF Chimera^28,29^ and excluded for analysis if close atomic contacts or missing polypeptide segments in the middle of a chain were found in the vicinity of the binding site.

**Figure 9:**
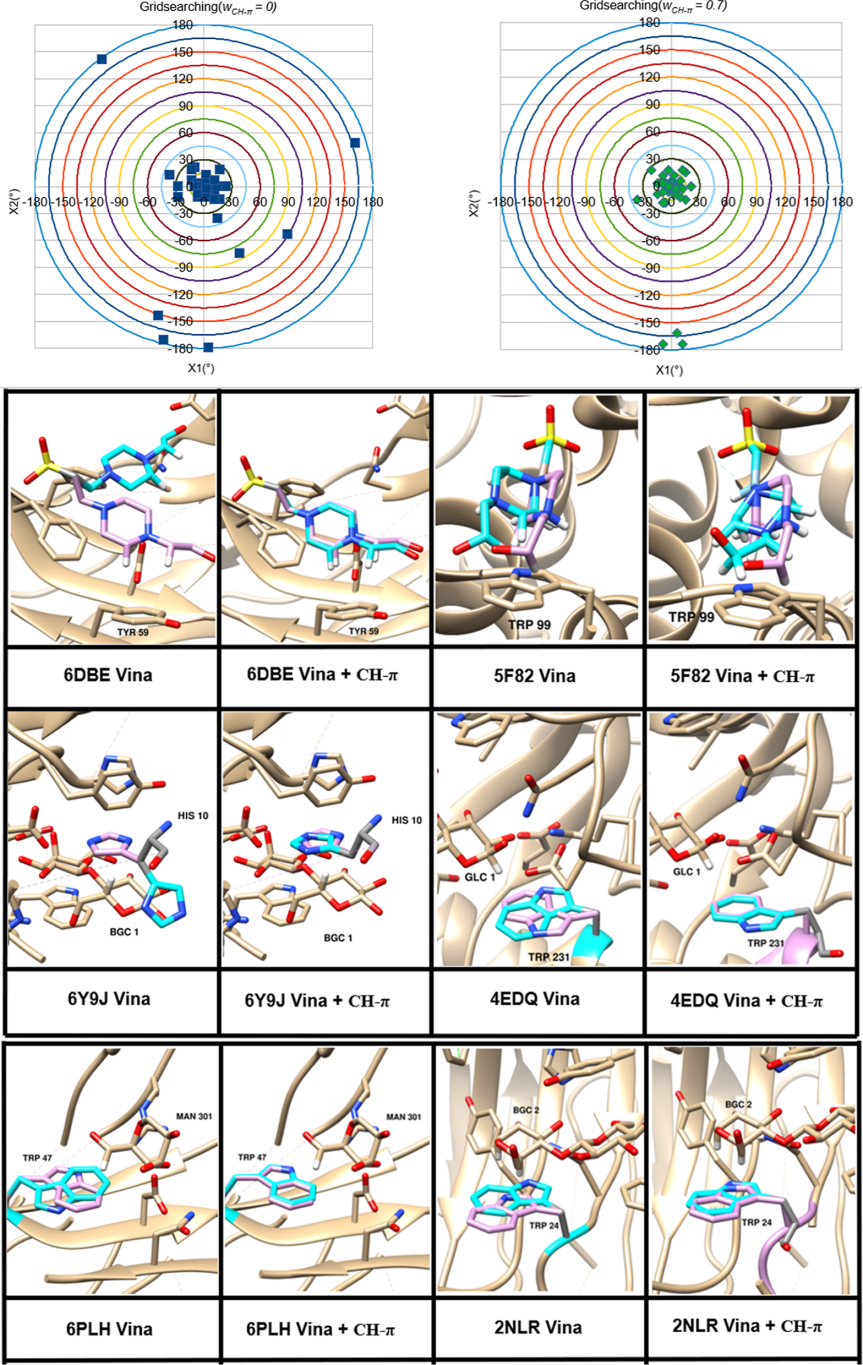
Top: bullseye plots illustrating the difference (°) between the torsional values of the rotated bonds in the top theoretical pose and that of the experimental structure. Only structures that demonstrated a change in the rmsd of at least 0.1 Å are shown (86 out of 228). The origin of the graph corresponds to theoretical structures that agree perfectly with the crystallographic data. (Left) Results from VINA without including any CH−π contribution (*w*_CH−π_ = 0). (Right) Results from VINA including the optimized CH−π contribution (*w*_CH−π_ = 0.7). Bottom: examples of structural predictions improved by inclusion of CH−π interactions. The protein backbone is shown in ribbon representation (beige). The experimental structure of the moiety displaying a CH−π interaction is shown in pink, while the predicted pose of that moiety is shown in cyan. The hydrogen atom of the aliphatic moiety involved in the interaction is shown in white.

Up to two rotatable bonds in the ligand or amino acid side chain were identified whose orientation defined the position of the moiety that formed the CH−π interaction in the complex. If the rotatable bonds were in the ligand (171 structures), they were selected to be those closest to the CH−π forming moiety. For amino acid side chains containing π groups (PHE, ARG, HIS, TYR, and TRP, 36 structures), the rotatable bonds were defined as the *N*-Cα–Cβ–Cγ and the Cα–Cβ–Cγ–Cδ1 torsion angle. If multiple residues in the same protein formed CH−π interactions with the ligand, each residue was treated as a unique instance (57 instances). A grid search (0 to 360°) of the *n* rotatable torsion angles was then performed (3° increment), resulting in a total of 120^*n*^ ligand or side chain conformations. To compensate for the lack of an internal energy component in the AD VINA scoring function, the following geometric criteria were employed to eliminate rotamers that resulted in severe intramolecular clashes: a separation of 2.0 Å or less between any two heavy atoms; a separation of 1.75 Å or less between any heavy atom and hydrogen atom; or a separation of 1.40 Å or less between any two hydrogen atoms. The total AD VINA interaction energy including the CH−π contribution was then computed for each moiety rotamer in the ligand or amino acid side chain; the rotamer resulting in the highest predicted affinity was thus determined. The weighting of the CH−π contribution (*w*_CH−π_, eq 3) was varied (between 0 and 1) to minimize the rmsd (Å) between the optimal theoretical rotamer and the experimentally determined structure, averaged over all the cocomplexes. The rmsd was highest (0.84 Å) at *w*_CH−π_ = 0.0, as expected, and rapidly decayed to a value of approximately 0.55 Å at *w*_CH−π_ = 0.3. Larger function weighting had only a modest improvement on the rmsd, with a minimum (0.49 Å) occurring at *w*_CH−π_ = 0.7 (Figure S4). Of the 228 total interactions (57 from protein side chain rotations, 171 from ligand moiety rotations, Table S4), the majority were insensitive to the presence of the CH−π contribution. Nevertheless, in six examples, the inclusion of the CH−π term markedly improved the orientation of the aromatic rings relative to the CH moiety (Figure 9). While this is a small fraction of the total number of structures, the improvement in the accuracy of the predicted poses in these cases is notable (Figure 9).

The structures that underwent the greatest improvement included 6DBE, 5F82, 6Y9J, 4EDQ, 6PLH, and 2NLR (Figure 9). In 6DBE, for example, the AD VINA scoring function predicted the presence of hydrogen-bond interactions between a hydroxyl group in the ligand and the side chains of nearby ASP and ASN residues, causing a significant deviation from the experimental structure. In contrast, inclusion of the CH−π term resulted in the correct prediction of an interaction between the aliphatic CH bonds in the ligand and TYR 59. In 5F82, the binding pose predicted by AD VINA was approximately 120° away from the experimental structure; however, using a value of *w*_CH−π_ = 0.7, the predicted pose was within 30° of the experimental structure. In this case, the inclusion of the CH−π term led to the correct prediction of an interaction between the aromatic side chain of TRP 99 and the aliphatic ligand group. In the 6Y9J example, the AD VINA scoring function placed the imidazole side chain of HIS 10 approximately 180° away from the observed orientation in order to introduce hydrogen bonding between the imidazole and the hydroxyl groups of the carbohydrate ligand. In contrast, inclusion of the CH−π term correctly prioritized the stacking between the imidazole ring and the aliphatic CH bonds on the carbohydrate ligand over these hydrogen-bonding interactions.

Similar improvements were seen for 4EDQ, 6PLH, and 2NLR in which a value of *w*_CH−π_ = 0.7 rescued the CH−π interactions associated with the indole side chain of TRP.

**Figure 10:**
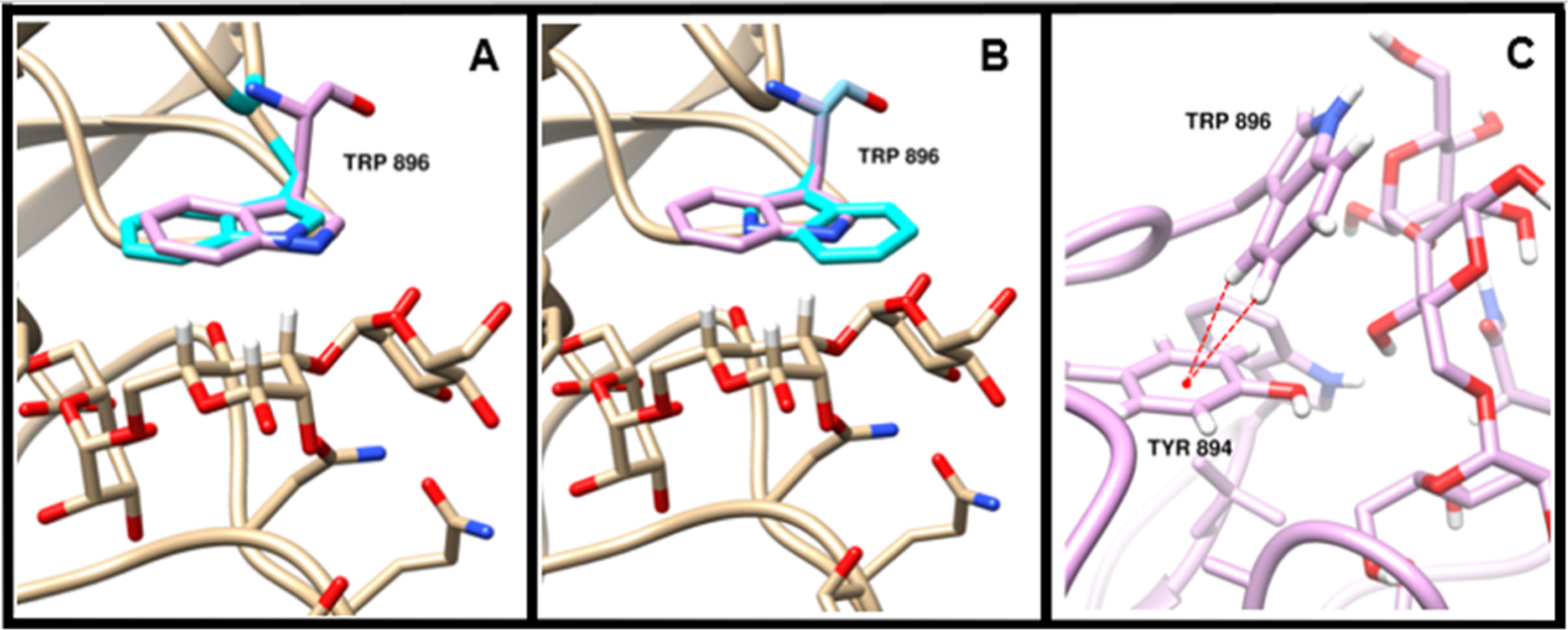
Incorrect prediction of TRP 896 in the 5V6F system. The protein backbone is shown in ribbon representation. In panels A (without CH−π) and B (with CH−π), the experimental structure of the aromatic moiety participating in the CH−π interaction is shown in pink, while the predicted pose of that moiety is shown in cyan. The hydrogen atoms of the aliphatic moiety are shown in white. In panel C, the experimental structure displays a potentially favorable CH−π interaction between TRP 896 and TYR 894.

The only structure that was significantly degraded by inclusion of the CH−π term (5V6F) is shown in Figure 10. In this complex, the difference between the observed and predicted orientation (rmsd = 0.82 Å with *w*_CH−π_ = 0.0, panel A and 3.33 Å with *w*_CH−π_ = 0.7, panel B) resulted from a flip of the indole ring of TRP 896. In this case, inclusion of the CH−π term resulted in an interaction energy of −16.7 kcal/mol for the experimental pose and −17.0 kcal/mol for the flipped orientation. It is notable that in the experimental structure, the indole ring forms a potentially favorable interaction with a neighboring tyrosine (TYR 894), an interaction that is lost when the indole ring is rotated in the grid searching process (Figure 10, panel C). Because interactions between receptor side chains are not included as part of the VINA scoring function, the loss of this interaction is not detected in the search for favorable interactions with the ligand. Thus, we do not believe that this erroneous structure prediction results from an overestimation of the strength of the CH−π term but rather from a lack of inclusion of the receptor internal energy.

### Hydrogen-Independent Functional Form and Docking Implementation

Unlike earlier versions of AutoDock, AD VINA does not consider hydrogen atoms in its scoring function. Thus, in order to incorporate CH−π interactions directly into AD VINA, a hydrogen-independent functional form was developed based solely on the interatomic distances (*R*_CC_) between the aliphatic carbon and ring aromatic carbons (Figure 5, right and eq 4). By removing the hydrogen-dependent angular component [*f*(θ_2_), eq 2], the carbon-only form (eq 4) is similar to equation [1] when *f*(θ_2_) has a value of unity. This is true only for complexes A, D, and E (Figure 6), which were thus selected for use in deriving the optimal parameters for eq 4.
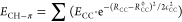
4where, “*E*_CC_” is the maximum interaction energy of each aliphatic carbon–aromatic carbon atom pair, “*R*_CC_” is the distance between that pair, “*R*^0^_CC_” is the optimal separation for that atom pair, and “*c*_CC_” controls the breadth of the curve.

**Figure 11:**
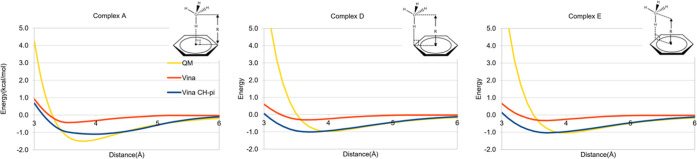
Methane–benzene dimer interaction energies at various vertical distances for three different dimer geometries (employed for deriving a hydrogen-independent function). When hydrogens are omitted, the six dimer geometries in Figure 6 reduce to three. Horizontal axis, C-centroid vertical distance (Å), vertical axis, interaction energy (kcal/mol). Yellow line, QM interaction energy, red line, energy from original AD VINA scoring function. Blue line, energy from AD VINA + CH−π.

Constants *E*_CC_, *R*^0^_CC_, and *c*_CC_ were derived by fitting eq 4 to QM data for complexes A, D, and E computed at the MP2/aug-cc-pVDZ//B3LYP/6-31G(d) level (Figure 11).

Despite the omission of the angular term, a set of parameter values for *E*_CC_, *R*^0^_CC_, and *c*_CC_ (Eqn. 1) were generated that led to acceptable fitting to the QM data for the methane–benzene complexes (*R*^0^_CC_ = 4.49, *c*_CC_ = 0.75, and *E*_CC_ = 0.26). It can be seen that for complex A, the fit to the QM data resulted in a good overall reproduction of the curve; however, the same was not true for complexes D and E. We attribute this to the soft quadratic repulsion term in AD Vina and to the absence of the aliphatic protons, which prevents reproduction of the QM atomic repulsions, leading to an underestimation of the C-to-ring separation.

**Figure 12:**
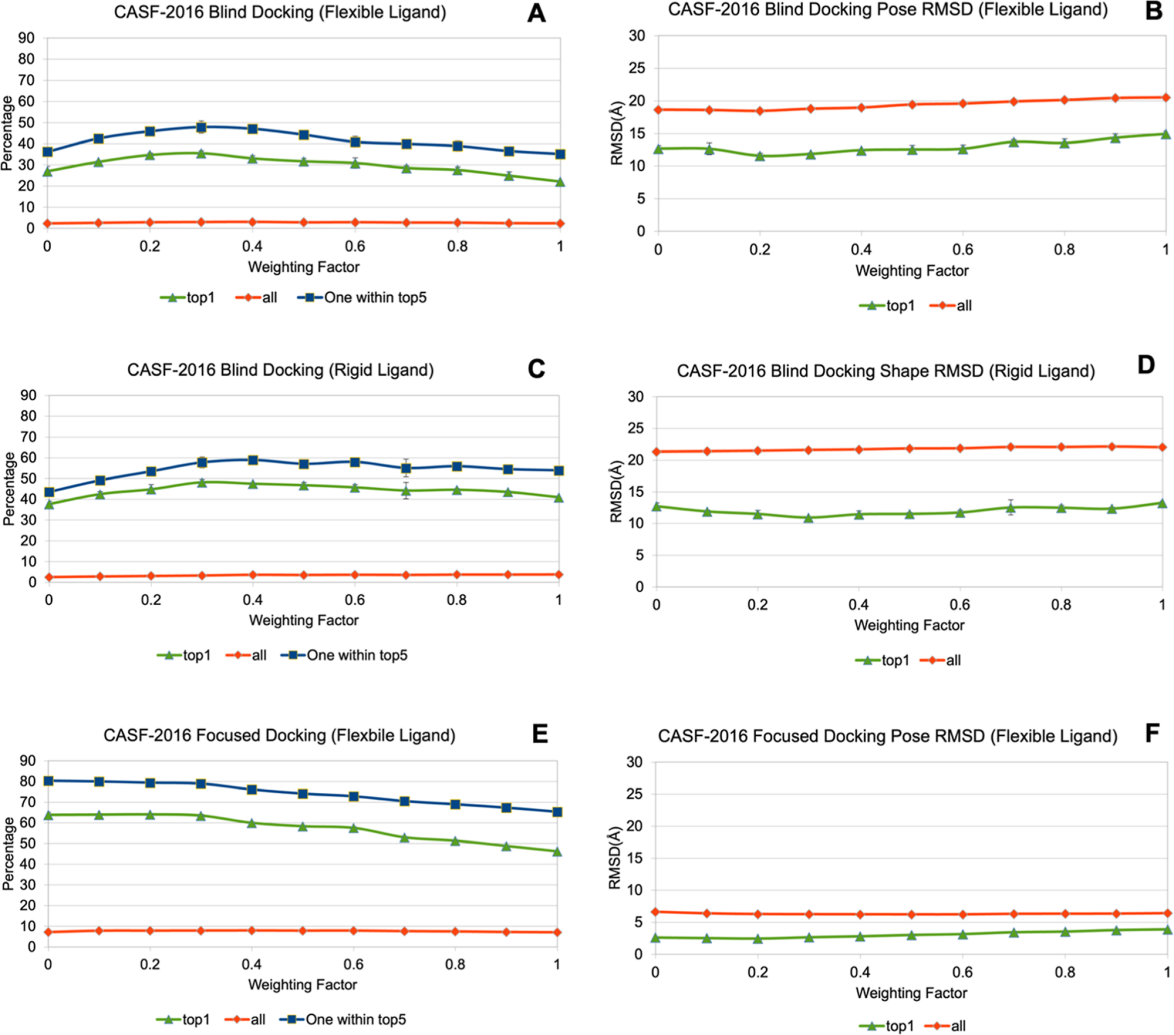
Docking performance of the CASF-2016 benchmark test set (*n* = 285). “Top 1” (green), the percentages of all top poses that were acceptable (ligand rmsd <2 Å to the experimental pose). “All” (red): the percentages of all docked poses that are acceptable; “One within top five” (blue): the percentages of all structures with at least one acceptable pose in the top five docked models. A: the various percentages from blind docking. B: the average rmsd values from blind docking. C: the various percentages from blind docking with the ligand held rigid in its experimental conformation. D: the average rmsd values from blind docking with the ligand held rigid in its experimental conformation. E: the various percentages from focused docking. F: the average rmsd values from focused docking.

The same geometric criteria employed for CH−π detection in PDB was applied on the CASF-2016^21^ benchmark set (*n* = 285), with 1213 CH−π interactions found among 265 structures (Table S5). On average, each of the 265 structures displayed 4.6 CH−π interactions confirming the ubiquity of CH−π interactions in protein-small complexes. In order to incorporate the hydrogen-independent CH−π term into AD-VINA, both blind and focused dockings were performed using the CASF-2016 benchmark set (Figure 12 and Table S6), while the weighting factor (*w*_CH−π_) for the CH−π term was varied from 0 to 1. Triplicate blind docking was performed with flexible and rigid ligands (all torsions frozen at experimental values), respectively, which allowed for the separation of errors originating from conformational sampling and scoring. A significant improvement in blind docking accuracy was observed in response to increasing *w*_CH−π_ up to 0.3 (Figure 12A,C). With flexible ligands (Figure 12A), the percentages of acceptable top-ranked poses (pose rmsd < 2 Å to experimental structure) increased from 27% with *w*_CH−π_ = 0.0 to 36% with *w*_CH−π_ = 0.3, and the percentages of structures with at least one acceptable pose among the top five ranked models increased from 36 to 48%. For focused docking (Figure 12E), a high level of accuracy (60–80%) was observed even in the absence of the CH−π term (*w*_CH−π_ = 0.0). Given the restricted search space centered on the ligand-binding site, little room for performance enhancement was available. Consequently, no improvement was observed with the inclusion of CH−π interactions up to *w*_CH−π_ = 0.3. Moreover, as the contribution of the CH−π term was increased beyond *w*_CH−π_ = 0.3, the accuracy of the predicted poses degraded monotonically. An optimal value of 0.3 for *w*_CH−π_ was therefore determined from the CASF-2016 examination. Given the importance of CH−π interaction in carbohydrate–protein complexes,^11−13^ we then examined the performance of this docking protocol on a curated data set culled from the PDB that included carbohydrate ligands (see Methods).

**Figure 13:**
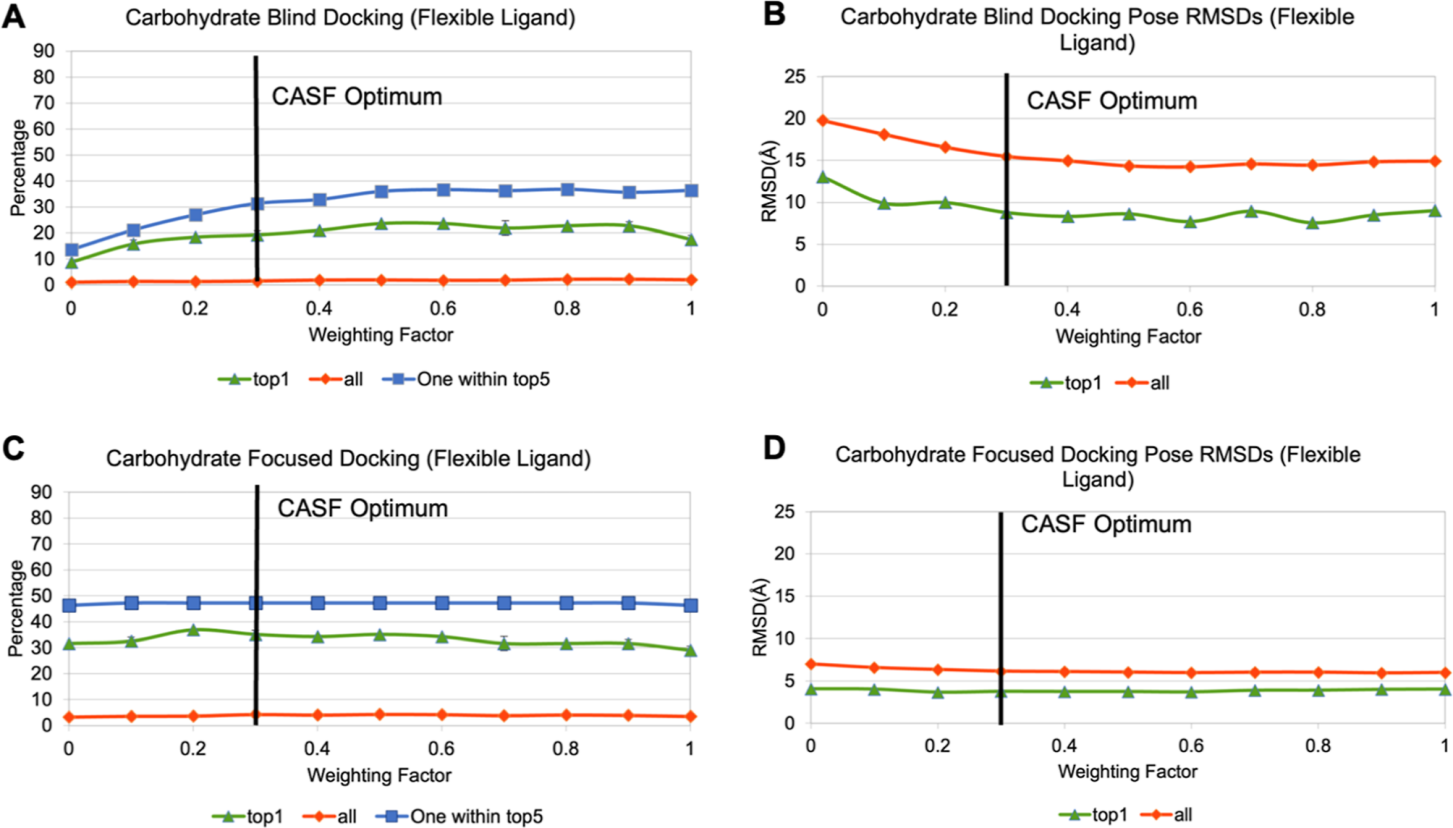
Percentage of acceptable poses and average pose-rmsd values from carbohydrate docking. Horizontal axis, CH−π weighting factor, vertical axis, percentage of acceptable poses or average pose-rmsd. Green, percentage of all top-ranked poses that are acceptable or average rmsd of all top-ranked poses. Red, percentage of all poses that are also acceptable or average rmsd of all poses. Blue, percentage of structures with at least one acceptable pose in the top five poses. (A) Blind docking percentages. (B) Blind docking pose rmsd values. (C) Focused docking percentages. (D) Focused docking pose rmsd values.

A total of 179 CH−π interactions were detected in the carbohydrate data set (*n* = 36), resulting in an average of 5.0 interactions per structure (Table S7). The hydrogen-independent functional form was also implemented in VINA-CARB,^16^ a variation of AD VINA augmented for carbohydrate docking. Both blind and focused redockings were performed in triplicate with VINA-CARB using the test set of 36 protein-carbohydrate crystal structures under varying values of *w*_CH−π_ (Figure 13 and Table S8). It can be observed that significant improvement was achieved by the functional form during blind docking (Figure 13). The percentage of structure with at least one acceptable pose within the top five (based on total score) increased from 18% (with no CH−π contribution, *w*_CH−π_ = 0.0) to 32% with a weighting factor of 0.8 (*w*_CH−π_ = 0.8). Similarly, the percentage of the top scoring poses that were acceptable nearly tripled from 9% (*w*_CH−π_ = 0.0) to 24% (*w*_CH−π_ = 0.8). Beyond a weighting of 0.8, the docking performance began to decrease, suggesting that the functional form was being overweighted from that point on.

Overall, with a weighting factor of 0.8, the functional form was able to decrease the average pose-rmsd of all models and all top models by approximately 5 Å during blind docking. On the other hand, a much more modest improvement was observed with focused docking as the base performance level with *w*_CH−π_ = 0.0 was significantly higher than that in blind docking (Figure 13). Unlike blind docking, the function did not increase the percentage of structures with at least one acceptable pose in the top five models in focused docking. The percentage of top structures that were acceptable increased by 5.6%, corresponding to only two additional structures on average. This modest improvement can again be observed by an improvement of approximately 1 Å for the average rmsd of all generated poses and approximately 0.5 Å for all top poses. Although *w*_CH−π_ = 0.8 appeared to be the optimal value for the carbohydrate data set, the docking performance was not significantly better than *w*_CH−π_ = 0.3, which was found to be optimal from the CASF-2016 examination. Similarly, the improvement in the performance of the rotamer grid searching study reached a plateau at approximately *w*_CH−π_ = 0.3. Given the relatively small sample sizes of the carbohydrate data sets and the limited benefit of employing *w*_CH−π_ > 0.3, we conclude that a general weighting of 0.3 should be applied to the CH−π function in AD VINA or VINA-CARB.

**Figure 14:**
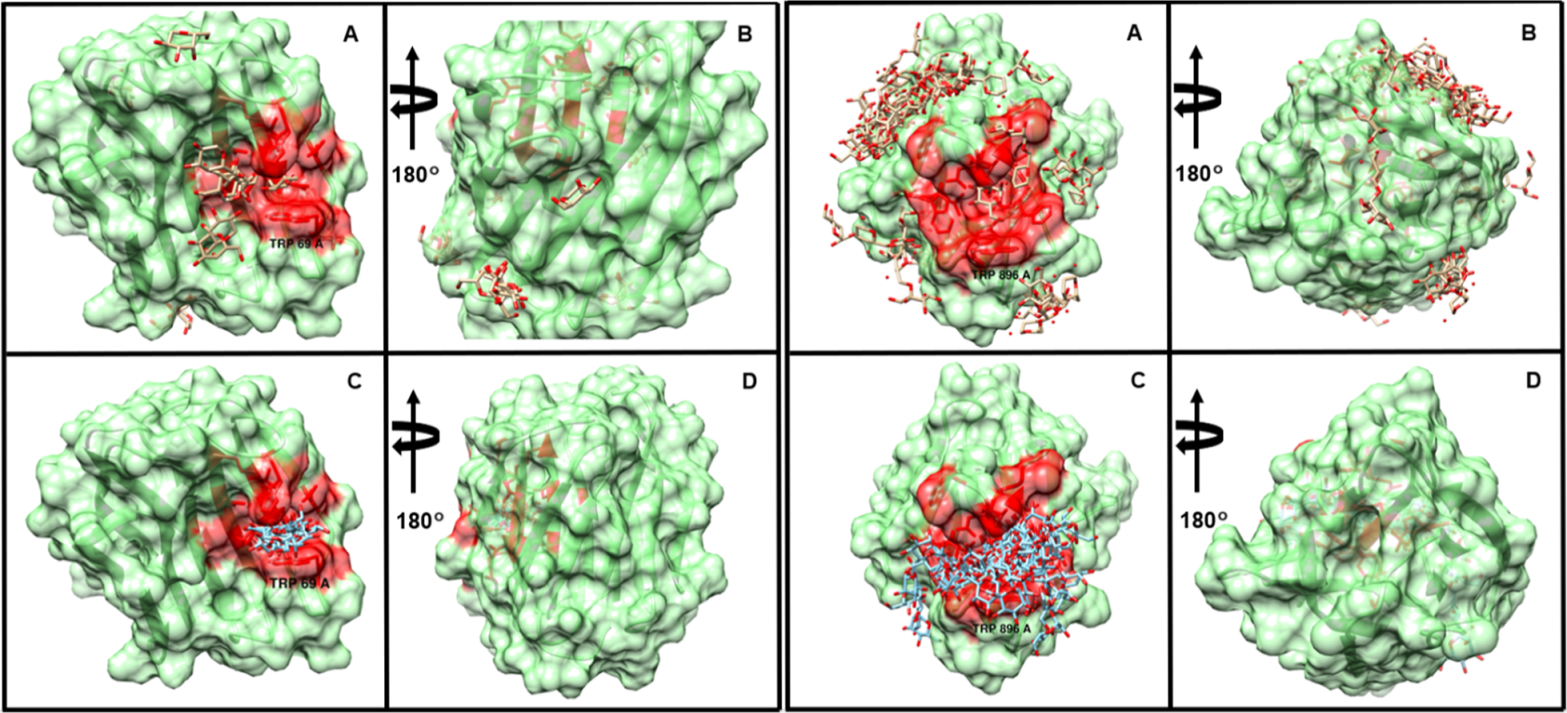
Two examples of blind docking with a random seed of 100,000 with the solvent accessible protein surfaces shown in green. Protein residues within 5 Å of the known ligand-binding site are colored red. Top, PDB ID 4NO4. Bottom, PDB ID 5V6F. (A) docked poses (beige) with no CH−π contribution. (B) A rotated by 180° around the vertical axis of the protein. (C) Docked poses (cyan) with *w*_CH−π_ = 0.8. (D) C rotated by 180° around the vertical axis of the protein. The protein residues involved in CH−π interactions with the ligand are labeled in each case; TRP 69 and TRP 896 for 4NO4 and 5V6F, respectively.

The pose-clustering effect of the functional form was clearly evident, for example, in the cases of PDB IDs 4NO4 and 5V6F from the carbohydrate data set (Figure 14). In each case, in the absence of any CH−π contribution, blind docking dispersed the ligands over the entire protein surface with little preference for the binding site. On the other hand, inclusion of CH−π (*w*_CH−π_ = 0.8) resulted in all docked poses being placed within a single cluster within the binding site (Figure 14). In 5V6F, without any CH−π contribution, the docked poses preferred to occupy shallow clefts on the protein surface, with only 1 out of 20 winding the actual binding site. Whereas with *w*_CH−π_ = 0.8, all poses were again concentrated in a single cleft that contains the actual binding site. In both cases, accounting for the protein-carbohydrate CH−π interactions, involving just a single TRP residue, resulted in significant improvement of blind docking performance.

## Conclusions

In summary, we have performed a structural study of nonredundant protein–ligand CH−π interactions in the PDB. The distribution of geometric patterns found in detected instances is in good agreement with previous theoretical studies^22,23,30,31^ as well as our own computations, suggesting that CH−π interactions are a significant driving force for aliphatic–aromatic interactions. A hydrogen-dependent empirical energy function was developed by fitting an empirical functional form to the results from QM calculations and structurally validated using a test set of 228 experimental complexes. Remarkably, the theoretical average interaction energies for complexes between aromatic amino acids and aliphatic moieties (−0.7 to −0.8 kcal/mol) were in excellent agreement with experimentally determined interaction energy (−0.80 to −0.86 kcal/mol) for PHE interacting with a monosaccharide.^27^ A hydrogen-independent energy function was subsequently developed for implementation within AD VINA (or AD VINA-CARB) that improved the ability of blind docking to correctly identify the bound ligand pose in complexes that contain CH−π interactions.

It can be observed that both grid searching and focused docking were frequently, but not completely, insensitive to the presence of the CH−π term (*w*_CH−π_ = 0.7). Nonetheless, it is important to note that inclusion of the CH−π term significantly improved the pose prediction for several structures (Figure 9) while only rarely degrading the quality of the prediction (Figure 10). It has been demonstrated that protein–ligand CH−π interactions are ubiquitous in the CASF-2016 benchmark set, which is representative of general protein-small-molecule binding events. The fact that CASF-2016 docking results were significantly improved by the functional form serves as a proof of concept that the CH−π interaction is not fully captured by van der Waals or dispersion interactions alone and that its general integration into typical automated molecular docking and simulation methods should be considered. Notably, in the case of carbohydrate blind docking, AD VINA-CARB was largely unable to correctly identify the carbohydrate-binding site, whereas when the contribution from CH−π interactions was included, a marked improvement in this ability was observed. It is interesting to note that, in contrast to blind docking, the performance of focused carbohydrate docking improved more modestly (from 60% with *w*_CH−π_ = 0.0 to 80% *w*_CH−π_ = 0.8).

It appears that, when performing focused redocking to a protein that was cocrystallized with the ligand, the complementarity of the protein and ligand shapes frequently, biases docking toward an acceptable pose regardless of the presence of the CH−π term. That explicit CH−π interactions are nevertheless a driving force in ligand recognition and are clearly evident from the blind docking examples (Figure 14), where their omission profoundly degraded the ability of AD VINA to detect an acceptable binding pose.

The observation that the experimental distribution of CH−π geometries does not show a preference for placing the CH bond above the ring centroid and perpendicular to the ring plane contrasts with expectations based on the QM data for the methane–benzene dimer. The origin of this discrepancy may be attributed to the observation that, while a canonical CH−π interaction is enthalpically favored, it is entropically disfavored,^32^ leading to a broader distribution of experimental interactions than that predicted from the methane–benzene analysis.

## Supporting Information Available

Detected CH−π instances in the PDB including methyl groups; detected CH−π instances in the PDB excluding methyl groups; CH−π instances; result data for grid searching analysis; detected CH−π instances in the CASF-2016 benchmark set; result data for CASF-2016 docking analysis; detected CH−π instances in the carbohydrate set; and result data for carbohydrate docking analysis (XLSX)
Histograms of the geometric quantities computed during CH−π detection; examples of CH−π interactions culled from the PDB involving the aromatic ring of PHE, HIS, TRP, and TYR, colored by AD VINA score (without CH−π); methane–benzene dimer interaction energy surfaces for the type B and C geometries at various vertical and horizontal distances and the corresponding histograms of the C-centroid horizontal offset values in the experimental structures; and rmsd (Å) versus empirical weighting factor (PDF)

## Notes

Download of the AD VINA–CH−π source code is available free of charge at GLYCAM-WEB (https://glycam.org/downloads/software/).
