# Supplementary material for: Protein–Ligand CH−π Interactions: Structural Informatics, Energy Function Development, and Docking Implementation
Source: J Chem Theory Comput. 2023 Jul 26;19(16):5503–15. doi: 10.1021/acs.jctc.3c00300 (PMC10448718; doi:10.1021/acs.jctc.3c00300)
Supplement: Supplementary file 2 — ct3c00300_si_002.pdf [file ct3c00300_si_002.pdf]

Supporting Information:

Protein-ligand CH-π interactions: structural-informatics, energy function development, and docking implementation

Yao Xiao and Robert J. Woods*

Complex Carbohydrate Research Center, University of Georgia, Athens, GA 30602, USA

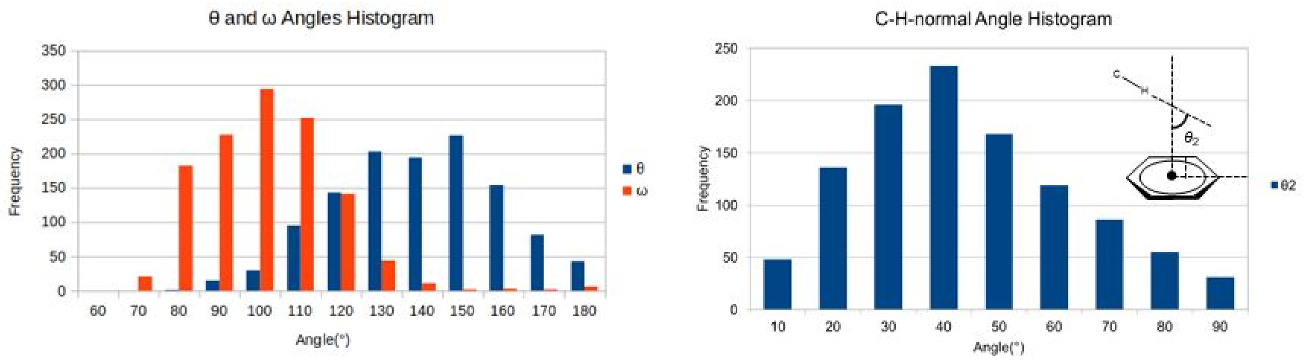

Figure S1. Histograms of the geometric quantities computed during CH-π detection. Left: histogram of the θ and ω. Right: histogram of C-H-normal angle. Horizontal axis: Distance(Å) or Angle (°). Each value on this axis represents the bin [previous value, this value). Vertical axis: frequency of occurrence of each bin.

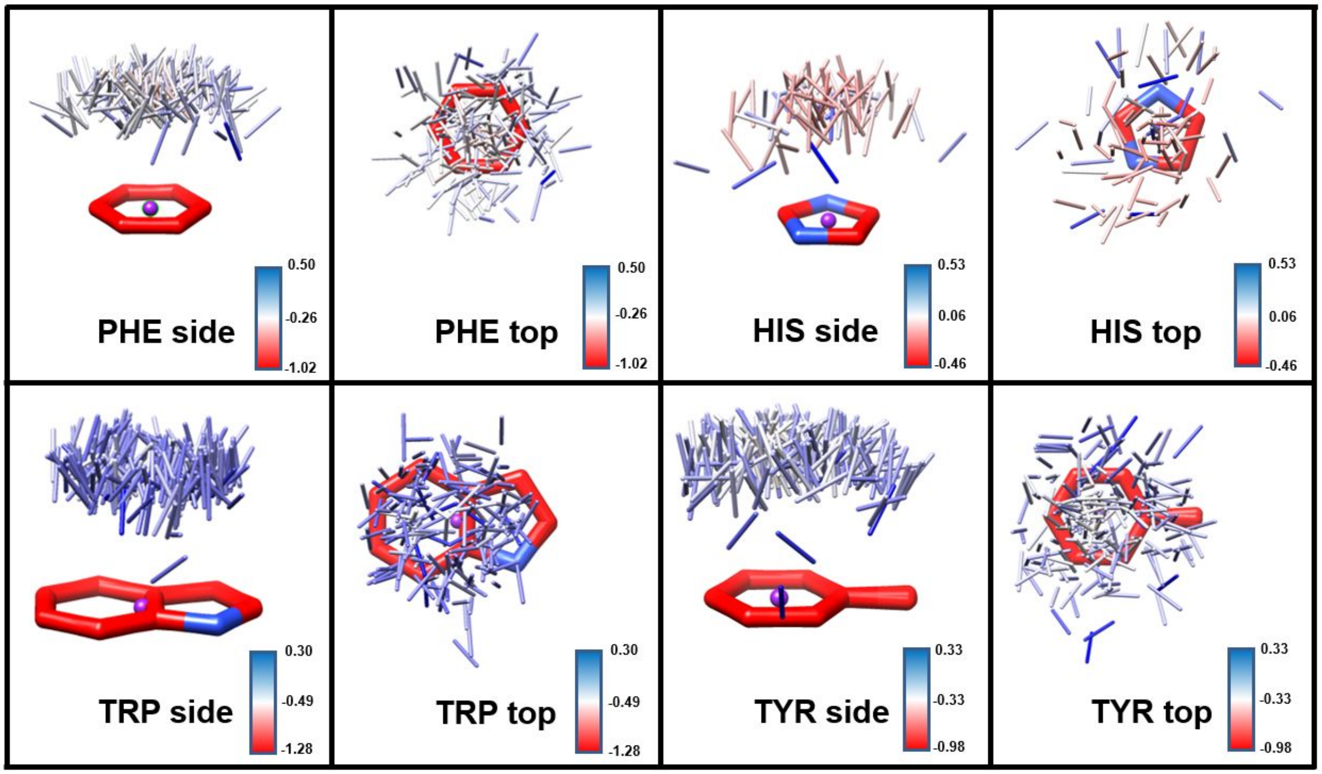

Figure S2. Examples of CH-π interactions culled from the PDB involving the aromatic ring of PHE, HIS, TRP, and TYR, colored by AD VINA score (without CH-π). A Red-white-blue color scale was employed, with red corresponding to the CH bond with the strongest interaction, white corresponding to zero interaction energy, and blue corresponding to the weakest interaction. Aromatic rings are uniformly colored in red, and the ring centroid purple. Each accompanying red-white-blue color gradient describes the range of interaction energies in that image.

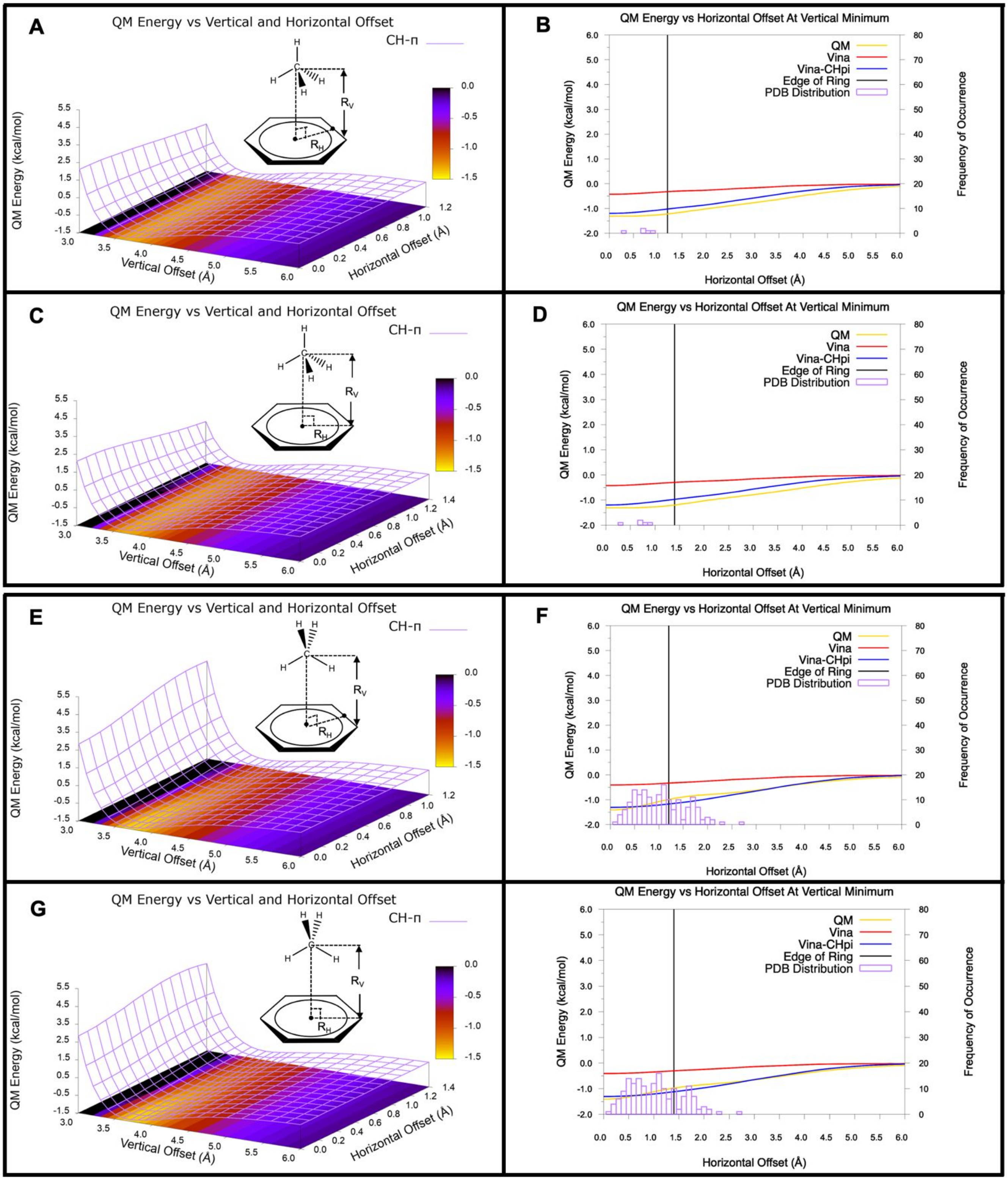

Figure S3. Methane-benzene dimer interaction energy surfaces for the type B and C geometries at various vertical and horizontal distances, and the corresponding histograms of the C-centroid horizontal

offset values in the experimental structures. A-D: type B geometry. A: QM energies of the dimer geometries with the methane translated horizontally along the vector between the ring centroid and the midpoint of a benzene C-C bond. B: QM energies of the dimer geometries as a function of horizontal offset (along ring centroid-bond midpoint), the corresponding VINA energies, VINA energies plus CH-π energies, and the C-centroid horizontal offset histogram corresponding to the type B geometry. C and D: same as A and B except that methane was horizontally translated along the radius of benzene. E-H: same as A-D but for the type C geometry.

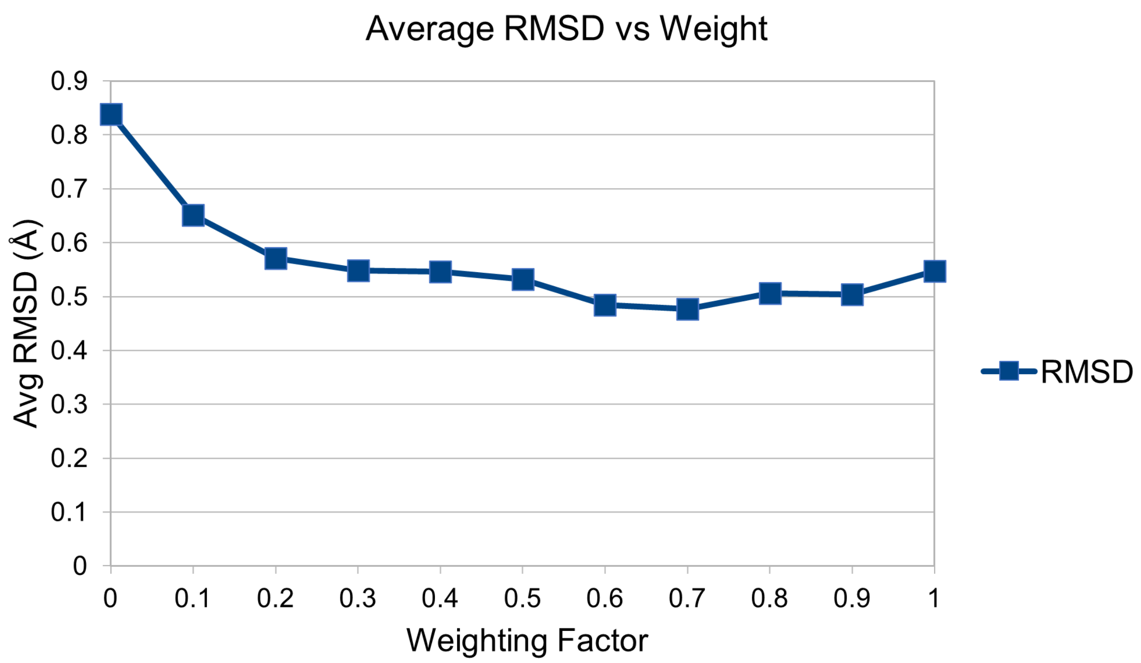

Figure S4. RMSD (Å) versus empirical weighting factor. Horizontal axis, value of weighting factor. Vertical axis, average RMSD of the test set.
